# Superiority of ^18^F-FAPI-42 PET/CT in the detection of primary tumor and management of appendiceal neoplasm to ^18^F-FDG PET/CT and CE-CT

**DOI:** 10.1186/s40644-024-00706-7

**Published:** 2024-05-07

**Authors:** Ye Dong, Shun Huang, Hubing Wu, Min Cao, Yanchao Huang, Ganghua Tang, Wenlan Zhou

**Affiliations:** 1 NanFang PET Center, NanFang Hospital, Southern Medical University, Guangzhou, Guangdong Province 510515 China; 2 GDMPA Key Laboratory for Quality Control and Evaluation of Radiopharmaceuticals, Department of Nuclear Medicine, Nanfang Hospital, Southern Medical University, Guangzhou, Guangdong Province 510515 China; 3 Department of Nuclear Medicine, PET Center, The Tenth Affiliated Hospital of Southern Medical University (Dongguan People’s Hospital), Dongguan, 523059 P. R. China

**Keywords:** Appendiceal neoplasm, ^18^F-FAPI-42, Contrast-enhanced CT, ^18^F-FDG, PET/CT

## Abstract

**Background:**

In the present study, we investigated the value of ^18^F-fibroblast-activation protein inhibitor (FAPI) positron emission tomography/computed tomography (^18^F-FAPI-42 PET/CT) to preoperative evaluations of appendiceal neoplasms and management for patients.

**Methods:**

This single-center retrospective clinical study, including 16 untreated and 6 treated patients, was performed from January 2022 to May 2023 at Southern Medical University Nanfang Hospital. Histopathologic examination and imaging follow-up served as the reference standard. ^18^F-FAPI-42 PET/CT was compared to ^18^F-fluorodeoxyglucose (^18^F-FDG) PET/CT and contrast-enhanced CT (CE-CT) in terms of maximal standardized uptake value (SUVmax), diagnostic efficacy and impact on treatment decisions.

**Results:**

The accurate detection of primary tumors and peritoneal metastases were improved from 28.6% (4/14) and 50% (8/16) for CE-CT, and 43.8% (7/16) and 85.0% (17/20) for ^18^F-FDG PET/CT, to 87.5% (14/16) and 100% (20/20) for ^18^F-FAPI-42 PET/CT. Compared to ^18^F-FDG PET/CT, ^18^F-FAPI-42 PET/CT detected more regions infiltrated by peritoneal metastases (108 vs. 43), thus produced a higher peritoneal cancer index (PCI) score (median PCI: 12 vs. 5, *P <* 0.01). ^18^F-FAPI-42 PET/CT changed the intended treatment plans in 35.7% (5/14) of patients compared to CE-CT and 25% (4/16) of patients compared to ^18^F-FDG PET/CT but did not improve the management of patients with recurrent tumors.

**Conclusions:**

The present study revealed that ^18^F-FAPI-42 PET/CT can supplement CE-CT and ^18^F-FDG PET/CT to provide a more accurate detection of appendiceal neoplasms and improved treatment decision making for patients.

## Background

Primary epithelial tumors of the appendix are a group of rare and heterogeneous neoplasms [1], including mucinous neoplasms and goblet cell carcinoma [2–4]. According to the new consensus statement in 2016 [5], mucinous neoplasms are classified as follows: adenoma, low-grade appendiceal mucinous neoplasms, high-grade appendiceal mucinous neoplasms, mucinous adenocarcinoma and poorly differentiated mucinous adenocarcinoma with signet ring features, while goblet cell carcinoma is a unique type of mixed endocrine-exocrine neoplasm.

At present, it remains challenging for imaging modalities to diagnose appendiceal neoplasms. Computed tomography (CT) is the most common imaging method used in the diagnosis and staging process of appendiceal neoplasms [6–8]. However, it is subject to some limitations, especially for the detection of primary tumors [9]. First, the appendix is a small organ and can be easily overlooked [10]. Second, it is sometimes difficult to detect the primary lesion through CT when the appendiceal neoplasm is small and adheres to the metastatic peritoneum, making it difficult to distinguish from the metastatic peritoneum [10]. Third, appendiceal neoplasms are also difficult to differentiate from acute appendicitis through CT imaging [11]. Therefore, in some patients, the diagnosis of appendiceal neoplasms cannot be identified until a laparoscopic exploration or surgery is performed.

^18^F-fluorodeoxyglucose (^18^F- FDG) PET/CT has been introduced into clinics as a supplement to CT in the diagnosis and staging of gastrointestinal tumors. However, for appendiceal neoplasms, ^18^F-FDG PET/CT has a great limitation because appendiceal neoplasms are often rich of mucus, which often present with low ^18^F-FDG uptake and lead to a low positive detection (approximately 35%) [12].

Radionuclide labelled, fibroblast-activated, protein inhibitors (FAPI), such as ^68^Ga-FAPI, have been developed as PET tracers and show superiority to ^18^F-FDG in imaging various cancers [13–15], especially in gastric cancer, pancreaticobiliary neoplasms and some rare tumors [16–18], although FAPI uptake can occur in non-oncologic conditions [19, 20]. It was found that gastrointestinal tumors, even those containing mucus or signet ring cell carcinoma, had high FAPI uptake and these lesions could be depicted clearly [14, 21]. A case report highlights the potential value of ^68^Ga-FAPI-04 PET/CT in visualization of appendiceal mucinous adenocarcinoma compared to ^18^F-FDG PET/CT [22]. Therefore, a hypothesis was established that FAPI PET/CT has some advantages in depicting appendiceal neoplasms compared to other imaging modalities. In the present study, a retrospective analysis was performed on 22 patients with appendiceal neoplasms to uncover the benefits of ^18^F-FAPI-42 PET/CT in detection of this tumor and disease management and compared those features to those of ^18^F-FDG PET/CT and CE-CT.

## Methods

### Patient selection

**Fig. 1:**
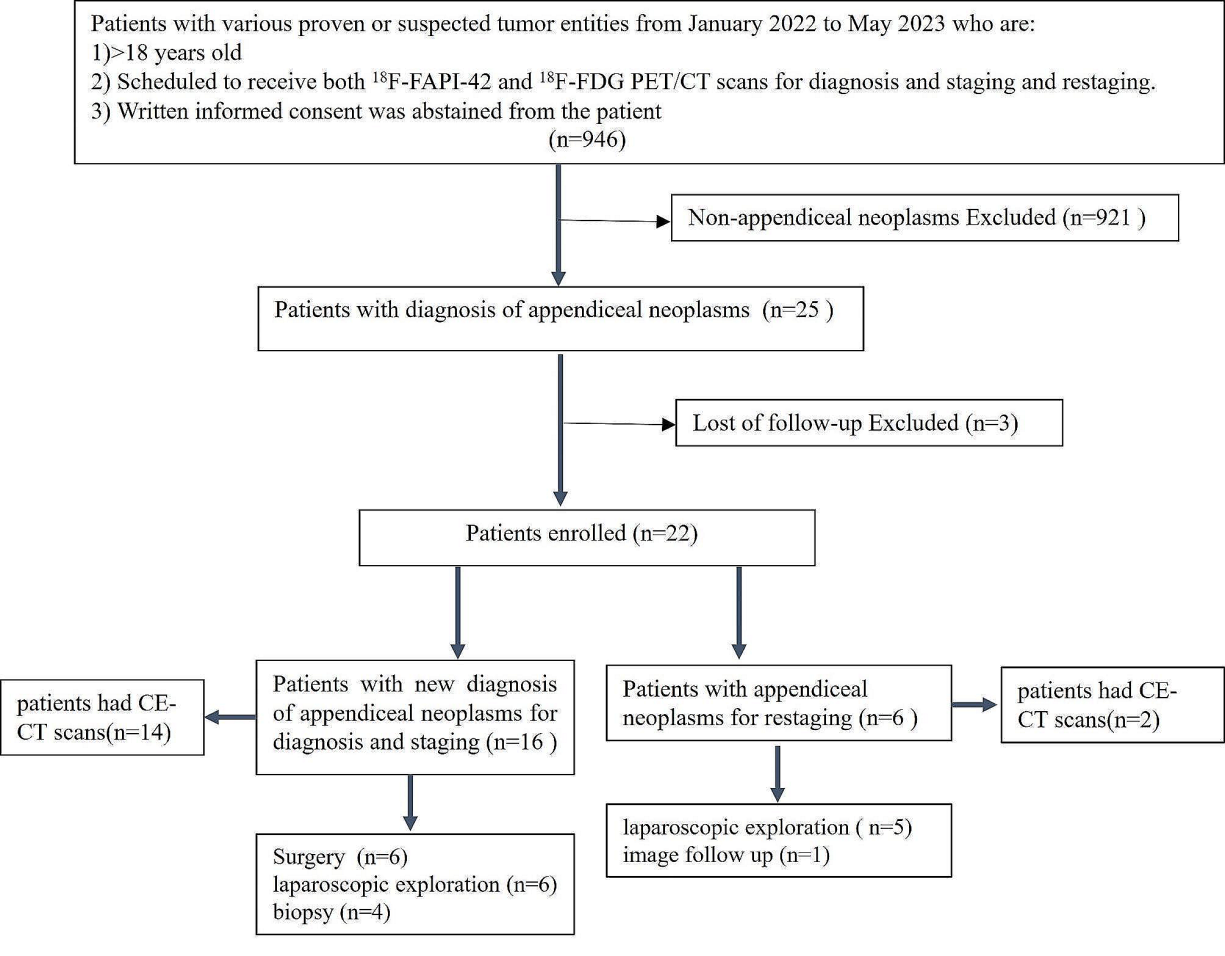
The flow chart summarizing of eligibility/exclusion criteria for the final study popular

This was a retrospective, single-center study obtained data from a prospectively acquired database approved by the Chinese Ethics Committee of Registering Clinical Trials and registered to the Chinese Clinical Trial Registry (ChiCTR2200059004). Written informed consent was obtained from all included patients. The retrospective analysis from January 2022 to May 2023 at Southern Medical University Nanfang Hospital focused on patients with appendiceal neoplasms and was conducted to evaluate the diagnostic performance and impact on clinical management of ^18^F-FAPI-42 PET/CT in comparison to ^18^F-FDG PET/CT and CE-CT. The flow chart summarizing the eligibility/exclusion criteria is shown in (Fig. 1). All patients were divided into either an initial group or a restaging group previously with appendiceal neoplasms who treated with chemo/radio/targeted therapy, and the time interval between completion of therapy and PET/CT scan being more than half a year (to avoid the treatment impact on radiotracer uptake). All patients completed imaging examinations within one month and did not receive anti-tumor treatment during this period. Histopathological findings obtained from biopsy or resected surgical specimens and imaging follow-ups were used as a reference for final diagnosis. Histopathology was obtained within 1 month after imaging examination. A total of 22 patients were included for clinical and imaging characteristics analysis.

### ^18^ F-FDG and ^18^ F-FAPI-42 Acquisition

^18^F-FAPI-42 was synthesized according to the previous article prior to injection [23]. ^18^F-FDG was automatically synthesized using a PET trace cyclotron (GE Healthcare) and the ^18^F-FDG synthesizer module Tracerlab FXF-N (Beijing PET Biotechnology Co. Ltd). The radiochemical purity of ^18^F-FDG and ^18^F-FAPI-42 exceeded 95%. All radiotracers were sterile and pyrogen-free to meet the criteria for human administration.

### PET/CT image acquisition

PET scans were performed on dedicated PET/CT scanners (Biograph mCTx scanner, Siemens Healthcare, Germany; uEXPLORER PET/CT scanner, United Imaging Healthcare, Shanghai, China) [24, 25]. The median time interval between FDG and FAPI PET/CT was 2 days (1–7 days). For ^18^F-FDG PET/CT scans, patients were instructed to fast for 4–6 h prior, to ensure a blood glucose level of ≤ 11.1 mmol/L at the time of tracer injection. For ^18^F-FAPI-42 PET/CT scans, patients were instructed to fast for about 2 h prior, to decrease hepatobiliary excretion before ^18^F-FAPI-42 tracer injection. ^18^F-FDG was administered at a median dose of 255 MBq (range: 191–378 MBq), and ^18^F-FAPI-42 tracer was administrated at a median dose of 153 MBq (range: 80–244 MBq). The time between injection and imaging was approximately 60 min for both modalities, followed by a whole-body PET/CT scan [26]. PET scans were acquired in 3D mode with a 5 min duration for total body of uEXPLORER and with a 2 min/bed position for whole body of BiographmCTx. Non-enhanced, low-dose CT was performed using a voltage of 120 KeV, current of 80 mA, and slice thickness of 2.0 mm. All data was reconstructed using OSEM-PSF-TOF.

### PET/CT image interpretation

Fused PET/CT images were reviewed on the MedEx system (MedEx Technology Limited Corporation) for registration, fusion, and measurement. Two experienced nuclear medicine physicians (YD and WL Zhou) assessed the PET/CT images, both of whom had more than 10 years of experience in nuclear oncology. Any non-physiologic uptake of ^18^F-FDG, or ^18^F-FAPI-42, greater than the adjacent normal tissue background for PET was considered a positive lesion. Any inconsistency between the two physicians was resolved by consensus. The PET/CT findings were grouped as primary tumor, lymph node metastasis and peritoneal metastasis. The patterns of peritoneal involvement were classified as diffuse type and nodular type. The peritoneal cancer index (PCI), established by Sugarbaker’s region, was used to evaluate the extent and severity of peritoneal metastases [27]. The standardized uptake values (SUV) of lesions were measured by the same nuclear medicine physician (YD) using the volume of interest method with the same standard. Paired SUVs of ^18^F-FAPI-42 and ^18^F-FDG were measured for comparison. The SUVmax of peritoneal metastasis was calculated according to the involved region, based on Sugarbaker’s 13 regions. Target-to-background rate (TBR) was calculated by dividing the SUVmax of the lesion by the mean SUV of the background (colorectal background for appendiceal neoplasms, abdominal fat space for lymph node and peritoneal lesions).

### Enhanced computed tomography image review

CE-CT was performed on 16 patients, with 14 at the pre-treatment phase and 2 in the post-treatment phase. The median time interval between CE-CT and FDG PET/CT was 7(3–14) days. CT images were reviewed by two physicians, including one senior physician who had more than 10 years of experience in CT diagnosis. Diagnostic results were collected from the Electronic Medical Record System (EMRS) of our hospital and classified into three levels: diagnosis of appendiceal neoplasms, suspect tumor but not originating from appendix, and benign lesion. When the CE-CT report considered an appendiceal tumor, it was considered positive, while other diagnoses were classified as negative.

### Management

The final diagnosis is based on a comprehensive evaluation of imaging examinations, pathological findings, and clinical follow-up, which is considered a reference standard. The management reference standard was the consensus of the multidisciplinary team (MDT) team in accordance with the final diagnosis and the National Comprehensive Cancer Network (NCCN) guidelines. Imaging guided management was compared with the reference standard. Treatment strategies for patients were classified either as diagnosis changed as treatment plans changed, and diagnosis changed but treatment plan remained unchanged. For example, if a patient was diagnosed with tuberculous peritonitis by CE-CT while diagnosed with an appendiceal tumor by PET/CT, the diagnosis of the patient would change and the treatment plan would also change accordingly. If a patient was diagnosed with colon cancer with peritoneal metastasis by CE-CT while PET/CT provided a diagnosis of appendiceal cancer with peritoneal metastasis, the diagnosis changed but the treatment plan did not change.

### Statistical analysis

All statistical analyses were conducted using the SPSS 22.0 software (IBM, Armonk, NY, USA). Normally distributed variables are expressed as means ± standard deviations and skewed variables as medians and range. The diagnostic efficacy of imaging was determined using the McNemar test. Differences in SUVmax and TBR between ^18^F-FDG and ^18^F-FAPI-42 were evaluated using paired t-test (normally distributed variables) or Wilcoxon signed-rank test (skewed variables). The same test was used to compare PCI-FDG and PCI-FAPI. Two-tailed p-values of less than 0.05 were considered statistically significant.

## Results

### Patients characteristics

Twenty-two patients (17 men, 5 women) with appendiceal neoplasms and a median age of 60 (54–67) years were included in this study (Table 1). Of the 22 patients, 16 (72.7%) were newly diagnosed and received PET/CT scans for diagnosis and staging, while the remaining 6 (27.3%) patients had already received treatment and PET/CT was performed for restaging. Among the 22 patients, 16 (72.7%) patients presented with abdominal discomfort and 6 patients had no complaints. Elevated levels of the tumor markers, carbohydrate antigen 199 (CA199), carbohydrate antigen 72 − 4 (CA72-4) and carcinoembryonic antigen (CEA) were found in 6 of 20 (30.0%), 11 of 20 (55.0%) and 11 of 21 (52.4%) patients, respectively. The final diagnosis was established by histopathology from surgery in 6 patients (27.3%), laparoscopic exploration in 11 patients (50%), endoscopic biopsy in 4 patients (18.2%) and follow-up examination in 1 patient (4.5%). Regarding histopathology, 5(22.7%) were mucinous adenocarcinomas, 6 (27.3%) were poorly differentiated carcinoma with or without signet ring cell carcinoma, 4 (18.2%) were goblet cell carcinoma, 5 (22.7%) were low-grade appendiceal mucinous neoplasms and 2 (9.1%) were high-grade appendiceal mucinous neoplasms.

**Table 1:** Summary of basic patients characteristics

Characteristic	value
No. of patients	22
**Age**	
Median Interquartile range	60 54–67
**Sex**	
female	5
man	17
**Clinical symptom**	
none	6
abdominal pain	6
abdominal distension	4
increased number of defecations	2
elevated tumor marker	4
**Tumor marker**	
CA199	
**(0–37)µ/ml**	14
> 37**µ/ml**	6
CA724	
**(0-6.9)µ/ml**	9
> 6.9**µ/ml**	11
CEA	
**(0–5)µ/ml**	10
> 5**µ/ml**	11
**Indication for PET/CT**	
diagnosis	16
restaging	6
**Diagnostic criteria**	
surgery	6
laparoscopic exploration	11
enteroscopic biopsy	4
imaging follow-up	1
**Histologic findings**	
mucinous adenocarcinoma	5
poorly differentiated carcinoma	4
poorly differentiated carcinoma with signet ring cell carcinoma	2
goblet cell adenocarcinoma	4
low-grade appendiceal mucinous neoplasms	5
high-grade appendiceal mucinous neoplasms	2

### Diagnostic performance of ^18^F-FAPI-42 and ^18^F-FDG PET/CT and CE-CT in primary tumors

**Fig. 2:**
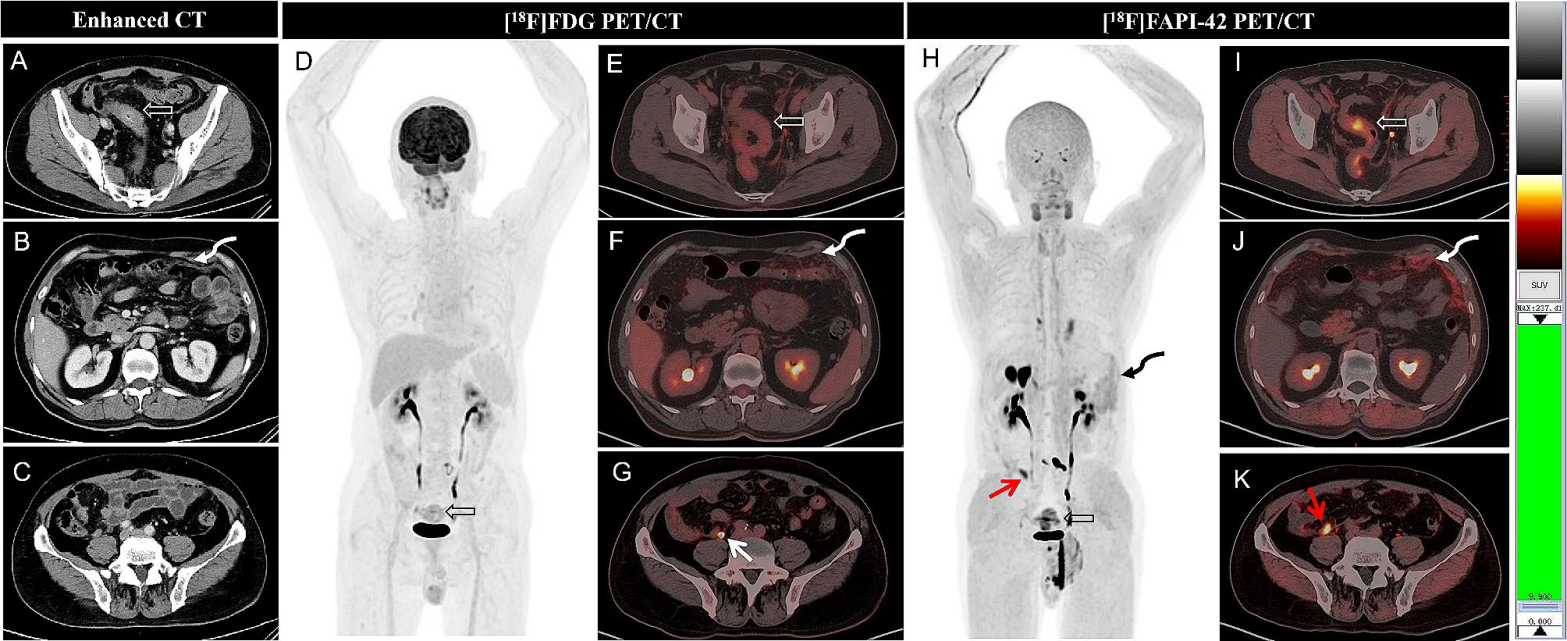
A 60-year-old man complained increased number of defecations, tumor markers of CA724 and CEA. The axial CE-CT showed thickening of the sigmoid colon wall (**A**, hollow arrow) and peritoneum in the left upper abdomen (**B**, bent arrow), suggested sigmoid colon cancer with peritoneal metastasis. The maximal intensity projection (MIP) of ^18^F-FDG PET/ CT showed mild ^18^F-FDG uptake in the pelvis (**D**, hollow arrow). The axial fused image PET/CT showed mild ^18^F-FDG uptake in the sigmoid colon (SUVmax, 3.0, **E**, hollow arrow) and omentum majus (SUVmax,1.6, **F**, bent arrow). The focus next to the appendix was physiologic uptake of ureter (**G**, white arrow). However, the MIP of ^18^F-FAPI-42 PET/ CT showed medium ^18^F-FAPI uptake in the lower right abdomen (**H**, red arrow), upper left abdomen (**H**, bent arrow) and sigmoid (**H**, hollow arrow). The axial fused image PET/CT showed moderate ^18^F-FAPI in the sigmoid (SUVmax, 5.4, **I**, hollow arrow) and omentum majus (SUVmax, 5.0, **J**, bent arrow), otherwise, the ^18^F-FAPI was also taken up by the lesions obviously in the appendix (SUVmax, 6.2, **K**, red arrow). Thus, the patient was diagnosed with appendiceal neoplasms and peritoneal metastasis which infiltrated the sigmoid colon. Then the patient underwent sigmoid puncture biopsy, which was diagnosed with goblet cell adenocarcinoma originating from the appendix. ^18^F-FAPI-42 PET/ CT was more sensitive to detect the primary tumor than ^18^F-FDG PET/ CT and CE-CT, although it did not change the clinical treatment plan

**Fig. 3:**
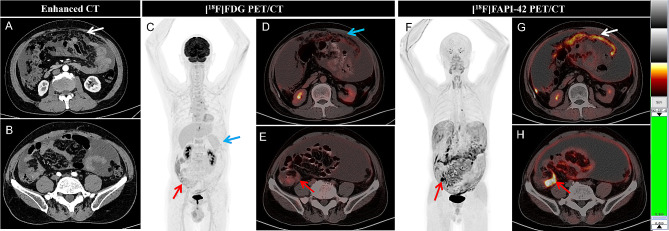
A 72-year-old man presented with abdominal distension for more than one month. The axial CE-CT suggested peritoneal tuberculosis (**A**, white arrow) and a large amount of ascites (**A**, **B**). ^18^F-FDG PET MIP showed no abnormal activity in the whole body (**C**), but the axial fused images showed mild ^18^F-FDG uptake in the omentum majus (SUVmax, 2.2, **D**, white arrow) and appendix (SUVmax, 4.7, **E**, red arrow). However, the MIP of ^18^F-FAPI-42 PET showed intense FAPI uptake in the abdominal (**F**, red arrow). The axial fused images showed intense FAPI uptake in the omentum majus (SUVmax, 11.8, **G**, white arrow) and appendix (SUVmax, 19.7, **H**, red arrow). Appendiceal neoplasms with peritoneal metastasis was diagnosed. Then the patient underwent abdominal exploration and was confirmed to be a poorly differentiated adenocarcinoma contained signet ring cell carcinoma by biopsy of omental node. ^18^F-FAPI-42 PET/ CT was more sensitive to detect the primary tumor and peritoneal metastasis than ^18^F-FDG PET/ CT and enhanced CT and changed the treatment plan compared to CE-CT

In the 16 newly diagnosed patients with appendiceal neoplasms, the mean diameters of 14 measurable primary lesions were 2.3 ± 0.7 (1.5–4.1) cm. ^18^F-FAPI-42 PET/CT detected lesions in 14 of 16 (87.5%) patients, while CE-CT detected lesions in 4 of 14 (28.6%) patients and ^18^F-FDG PET/CT detected lesions in 7 of 16 (43.8%) patients (Table 2). ^18^F-FAPI-42 PET/CT had a higher positive detection rate than CE-CT (87.5% vs. 28.6%; χ^2^ = 0.933, *P* = 0.008) (Figs. 2 and 3). It also had a trend to be higher than ^18^F-FDG PET/CT in detection of appendiceal neoplasms (87.5% vs. 43.8%), although the difference did not reach the significant level (χ^2^ = 1.778, *P* = 0.16). In 2 of 16 (12.5%) newly diagnosed patients, all three imaging modalities were negative for tumors, caused by appendiceal neoplasm ruptures. Compared to ^18^F-FDG PET/CT, ^18^F-FAPI-42 PET/CT had higher tracer uptake and TBR in primary tumors and imaged them more clearly (mean SUVmax, 8.3 vs. 3.3, *P* = 0.002; mean TBR, 13.1 vs. 2.8, *P* < 0.001) (Table 3; Figs. 2 and 3). Among the pathological subtypes of appendiceal neoplasms, lesions with low-grade appendiceal mucinous neoplasms/high-grade appendiceal mucinous neoplasms were observed to have lower ^18^F-FDG and ^18^F-FAPI-42 uptake than other pathological subtypes (all *P* < 0.05) except GCA in ^18^F-FDG (Table 4).

**Table 2:** Detection of primary lesion and peritoneal metastases by CE-CT, ^18^F-FDG PET/CT and ^18^F-FAPI-42 PET/CT

Indication	primary tumor + -		Positive detection rate	peritoneal metastases + -		Positive detection rate
CE-CT	4	10	28.6%	8	8	50.0%
^18^F-FDG PET/CT	7	9	43.8%	17	3	85.0%
^18^F-FAPI-42 PET/CT	14	2	87.5%	20	0	100%

**Table 3:** Comparison of tracer uptake in the lesions between ^18^F-FAPI-42 and ^18^F-FDG PET/CT

Tumor Lesions and Parameter	^18^F-FDG PET/CT	^18^F-FAPI-42 PET/CT	*P* Value
**Primary tumors**			
Positive Lesion Number	7	14	
Mean SUVmax Mean Background Mean TBR	3.3 ± 1.9 1.2 ± 0.4 2.8 ± 1.3	8.3 ± 6.2 0.6 ± 0.2 13.1 ± 9.2	0.002 < 0.001 < 0.001
**Positive lymph nodes**			
Lesion Number	13	29	
Mean SUVmax Mean TBR	2.3 ± 0.9 5.2 ± 5.1	7.3 ± 2.0 11.0 ± 7.8	< 0.001 < 0.001
**Positive peritoneal lesions**			
Involved regions number#	43	108	
Mean SUVmax Mean TBR	2.7 ± 1.6 5.6 ± 4.5	6.3 ± 3.5 14.0 ± 10.0	< 0.001 < 0.001

FAPI = fibroblast-activation protein inhibitor, ^18^F = fluorine 18, SUVmax = maximum standardized uptake value, TBR = target-to-background rate, # Calculated by involved regions according to the Sugarbaker’s region and SUVmax is obtained by measuring each involved area

**Table 4:** Comparison of ^18^F-FAPI-42 and ^18^F-FDG uptake in different pathological subtype of primary tumor and peritoneal metastases

		SUVmax			TBR		
Indication	No	FDG	FAPI	P*	FDG	FAPI	P*
**primary tumor**							
AD	5	4.7 ± 1.5	14.6 ± 3.2	0.003	3.7 ± 1.5	20.9 ± 6.8	0.006
MA	3	4.4 ± 2.3	9.8 ± 7.0	0.274	3.0 ± 1.4	14.6 ± 9.2	0.153
GCA	2	1.5 ± 0.8	7.9 ± 2.4	NA	1.8 ± 0.7	12.8 ± 11.3	NA
L/HAMN	5	2.0 ± 0.7	2.3 ± 1.0	0.642	2.0 ± 0.6	3.6 ± 1.3	0.095
***P*** ^***#***^		0.01	0.002		0.119	0.003	
**peritoneal metastases**							
AD	4	2.1 ± 0.9	6.5 ± 3.0	< 0.001	4.2 ± 3.4	16.9 ± 10.1	< 0.001
MA	7	3.3 ± 2.0	7.8 ± 4.0	< 0.001	7.1 ± 5.4	15.8 ± 11.7	< 0.001
GCA	4	2.0 ± 0.8	4.8 ± 1.9	< 0.001	4.4 ± 2.5	10.7 ± 6.5	< 0.001
L/HAMN	5	3.2 ± 1.7	5.2 ± 2.7	< 0.001	6.4 ± 5.0	11.5 ± 7.4	< 0.001
***P*** ^***#***^		0.611	0.89		0.742	0.563	

AD, adenocarcinoma; MA, mucinous adenocarcinoma; GCA, goblet cell adenocarcinoma; L/HAMN, low/high-grade appendiceal. mucinous neoplasms; NA: not applicable. P* stand for the comparison of FDG and FAPI uptake (SUVmax or TBR) of primary tumors or peritoneal metastases in the same pathological subtypes. *P*^#^ stands for the comparison of FDG or FAPI uptake (SUVmax or TBR) of primary tumors or peritoneal metastases among different pathological subtypes

### Diagnostic performance of ^18^F-FAPI-42, ^18^F-FDG PET/CT and CE-CT in peritoneal metastases

Peritoneal metastases occurred in 20 of 22 patients, including 15 newly diagnosed patients and 5 post-treatment patients. The distribution of peritoneal metastases presented as diffuse infiltration in 16 patients and nodular infiltration in 4 patients. Eight of 16 (50.0%) patients were diagnosed with peritoneal metastases by CE-CT, however, more patients with peritoneal metastases were detected by ^18^F-FDG PET/CT (17/20, 85.0%) and by ^18^F-FAPI-42 PET/CT (20/20, 100%) (Table 2). Compared to ^18^F-FDG PET/CT, ^18^F-FAPI-42 PET/CT detected more involved regions of peritoneal metastases (108 vs. 43) based on Sugarbaker’s 13 regions (Table 3).

**Fig. 4:**
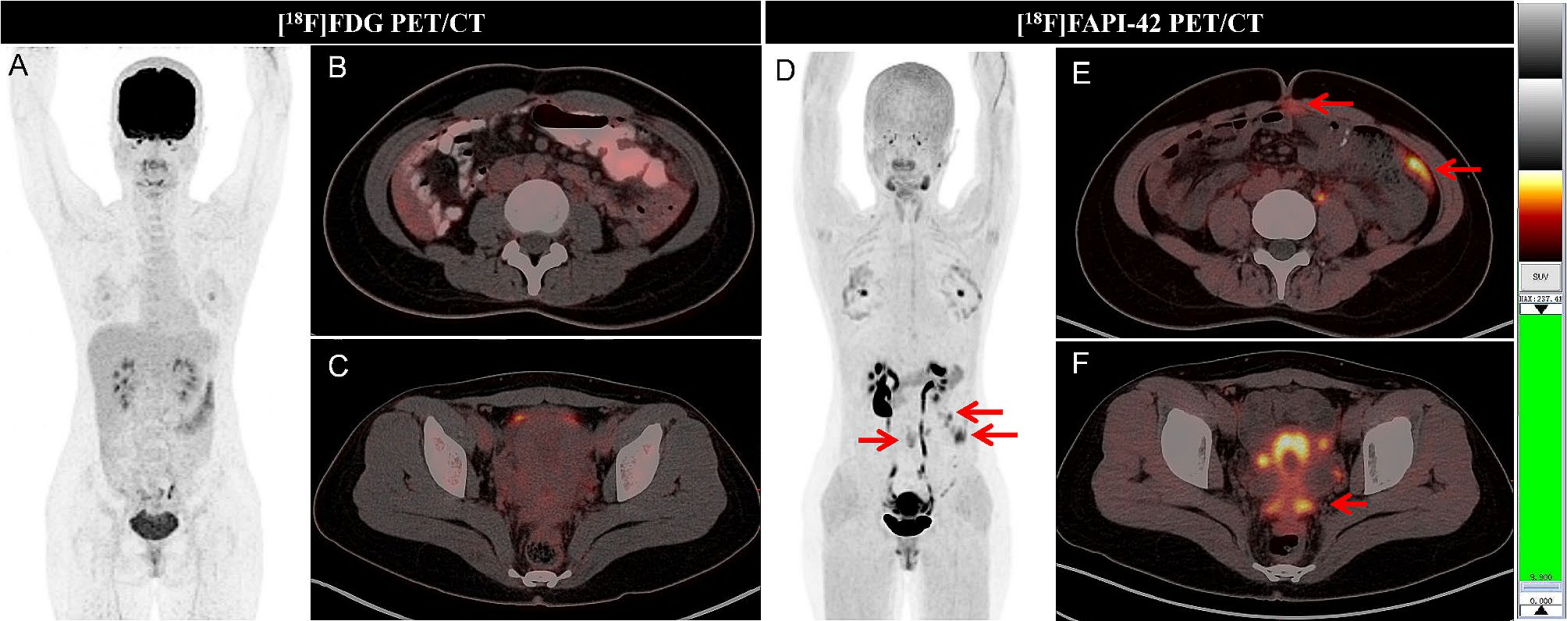
A 29-year-old woman had a medical history of surgery of goblet cell adenocarcinoma for 2 years and without discomfort. She underwent PET/CT scan for regular follow-up. However, no positive was observed on ^18^F-FDG PET MIP(A) and axial fused images (**A** ∼ **C**). However, the MIP of ^18^F-FAPI-42 PET showed mild to intense FAPI uptake in the middle and left abdominal (**D**, red arrows), the axial fused images of ^18^F-FAPI-42 PET/CT showed FAPI was taken up by the lesions in the left abdominal omentum majus (SUVmax, 3.4 ~ 5.7, **E**, red arrows) and utero-rectal recess (SUVmax, 8.2, **F**, red arrow).^18^F-FAPI-42 PET/CT is superior to ^18^F-FDG PET/CT in monitoring tumor recurrence in goblet cell carcinoma patient and changed the treatment plan

For visual analysis, ^18^F-FAPI-42 PET/CT imaged peritoneal metastases more clearly than ^18^F-FDG PET/CT in all 20 patients. Higher uptake and higher signal contrast of ^18^F-FAPI-42 in peritoneal metastases were observed compared to ^18^F-FDG (mean SUV max: 6.3 vs. 2.7, *P* < 0.001; mean TBR: 14.0 vs. 5.6, *P* < 0.001) (Table 3; Figs. 3 and 4). The median peritoneal cancer index (PCI) score derived from ^18^F-FAPI-42 PET/CT was higher than ^18^F-FDG PET/CT (median PCI: 12 vs. 5, *P* < 0.001) (Table 5). There is no statistically significant difference in the uptake of FDG or FAPI in peritoneal metastases among different pathological subtypes (all *P >* 0.05) (Table 4).

**Table 5:** Comparison of ^18^F-FDG and ^18^F-FAPI-42 PET/CT for PCI of peritoneal metastases in 20 patients

pathological subtype	No. of patients	PCI-FDG Median (range)	PCI-FAPI Median (range)	*P* value
AD	4	3(0–5)	18(2–28)	0.068
MA	7	8(0–13)	12(2–21)	0.018
GCA	4	5(2–10)	11(5–20)	0.042
L/HAMN	5	3(0–4)	10(6–14)	0.066
*P*	20	5(0–13)	12(2–28)	< 0.001

AD, adenocarcinoma; MA, mucinous adenocarcinoma; GCA, goblet cell adenocarcinoma;

L/HAMN, low/high-grade appendiceal mucinous neoplasms; PCI: peritoneal cancer index

### Other metastases

Of all 22 patients, 7 had lymph node metastases. ^18^F-FAPI-42 PET/CT detected more positive lymph nodes than ^18^F-FDG PET/CT (29 vs. 13). Higher ^18^F-FAPI-42 uptake occurred in these suspected lymph nodes compared to ^18^F-FDG (mean SUV max: 7.3 vs. 2.3, *P* < 0.001; mean TBR: 11.0 vs. 5.2, *P* < 0.001) (Table 3). Other sites infiltrated by appendiceal neoplasms were found in the pleura, sigmoid colon, rectum and seminal vesicle gland. Intense uptake ^18^F-FAPI-42 was also observed in all these lesions, while ^18^F-FDG PET/CT had only slight FDG uptake of lesions in the pleural and sigmoid.

### Changes in patients management

#### ^18^ F-FAPI-42 PET/CT vs. CE-CT in initially diagnosed patients

Of the 16 initially diagnosed patients, 14 underwent CE-CT scans. According to the CE-CT reports, 4 (28.6%) patients were diagnosed with appendiceal neoplasms, 3 (21.4%) were diagnosed with colon cancer with peritoneal metastases (Figs. 2), 2 (14.3%) were diagnosed with pseudomyxoma peritonei (PMP) with unknown origin, and 5 (35.7%) were diagnosed with benign diseases (e.g., liver cirrhosis, tuberculous peritonitis, and chronic pancreatitis with pseudocysts) without abdomen tumors (Fig. 3). However, of these 14 patients, 12 were diagnosed with appendiceal neoplasms with peritoneal metastasis by ^18^F-FAPI-42 PET/CT, one was diagnosed with PMP, and another had peritoneal metastasis with unknown origin. According to the results, ^18^F-FAPI-42 PET/CT changed the treatment decisions in 35.7% (5/14) of patients compared to CE-CT, and were originally diagnosed with benign diseases. While ^18^F-FAPI-42 PET/CT helped with detecting the origin tumors in 28.6% (4/14) patients, 3 were diagnosed with colon cancer and one with PMP by CE-CT.

#### ^18^ F-FAPI-42 PET/CT vs. ^18^ F-FDG PET/CT in initially diagnosed patients

Of 16 initially diagnosed patients, 4 (25.0%) were falsely diagnosed to have benign diseases without abdomen tumors by ^18^F-FDG PET/CT, including 2 patients diagnosed with liver cirrhosis and tuberculous peritonitis and 2 diagnosed with appendiceal cyst. However, all 4 patients were accurately diagnosed to be appendiceal neoplasms by ^18^F-FAPI-42 PET/CT and the treatment plan was changed as a result (Fig. 3). In 3 (18.8%) patients who were diagnosed to have peritoneal metastases with unknown origin, ^18^F-FAPI-42 PET/CT accurately detected the origin tumors although their treatment plans were not changed.

### Classic case

The patient in Fig. 3 was a 72-year-old man who presented with abdominal distension for more than one month and axial CE-CT suggested peritoneal tuberculosis. ^18^F-FDG PET/CT suspected peritoneal metastasis but could not find the primary tumor. ^18^F-FAPI-42 PET considered appendiceal neoplasms with peritoneal metastasis. This was followed by abdominal exploration which confirmed poorly differentiated adenocarcinoma containing signet ring cell carcinoma, determined by biopsy of the omental node. In this case, ^18^F-FAPI-42 PET/CT was more sensitive to primary tumor detection and peritoneal metastasis than ^18^F-FDG PET/CT and CE-CT and more frequently changed the treatment plan compared to CE-CT.

### ^18^ F-FAPI-42 PET/CT vs. ^18^ F-FDG PET/CT vs. CE-CT in restaging appendiceal neoplasm

In the restaging group, 5 of 6 patients were diagnosed with peritoneal metastases by both ^18^F-FAPI-42 PET/CT and ^18^F-FDG PET/CT. In 2 patients, peritoneal metastases were also found by CE-CT. ^18^F-FAPI-42 PET/CT did not change the treatment plan for these patients.

## Discussion

It is challenging for clinicians to diagnose appendiceal neoplasms before surgery [10]. Our study demonstrates that appendiceal neoplasms are a FAPI avid tumor. ^18^F-FAPI-42 PET/CT showed its superiority to ^18^F-FDG PET/CT and CE-CT in detection and visualization of primary tumors and metastases. It enhances the proportion of primary tumor detection from 28.6% of CE-CT and 43.8% of ^18^F-FDG PET/CT to 87.5%. It also affected treatment plans in 25.0% (4/16) and 35.7% (5/14) of patients compared to ^18^F-FDG PET/CT and CE-CT in patients initially diagnosed by other imaging modalities. Thus, ^18^F-FAPI-42 PET/CT may provide a new and beneficial imaging method in diagnosis and management for patients with appendiceal neoplasms.

Our data confirmed that CE-CT has limitations to its sensitivity of detecting appendiceal neoplasms and accurately differentiating appendiceal neoplasms from other diseases. It provided accurate diagnoses in only 28.6% of patients in the present study. Although ^18^F-FDG PET/CT had a higher positive detection rate (43.8%), it is not a satisfactory result for the clinic, which is mainly due to low uptake of ^18^F-FDG in the tumor (mean SUVmax, 3.3). The results of this study showed that ^18^F-FAPI-42 PET/CT may be a good modality for detection and diagnosing appendiceal neoplasms. High uptake of ^18^F-FAPI-42 (mean SUVmax, 8.3) in appendiceal neoplasms contributed to a high positive detection rate (87.5%). High uptake of ^18^F-FAPI-42 (mean SUVmax, 9.8) was even found in tumors rich of mucus, which always uptake ^18^F-FDG poorly and is the main cause of false negatives by ^18^F-FDG PET/CT. A similar phenomenon was reported where an intense uptake of FAPI occurred by mucinous tumors and gastric signet ring cell carcinomas in gastrointestinal tumors, which also uptake ^18^F-FDG poorly [28–30]. Our study indicated that ^18^F-FAPI-42 PET/CT may provide a new and exceptional diagnostic method for appendiceal neoplasms before treatment. However, the present study also implied that false negatives on ^18^F-FAPI-42 PET/CT may occur in some tumors with low invasiveness, such as low-grade appendiceal mucinous neoplasms and high-grade appendiceal mucinous neoplasms, where lower FAPI uptake (mean SUVmax only 2.3 ± 1.0) was observed.

The appendix is a small organ. When its cavity is filled with mucus, the appendix will rupture which leads to peritoneal diffuse metastasis [31, 32], therefore, peritoneal metastasis is commonly seen in patients with appendiceal neoplasms. It is crucial to accurately evaluate the peritoneal status to decide whether radical surgery is feasible [33, 34]. In this present study, although peritoneal thickening and pseudomyxoma peritonei could be observed by CE-CT, it was always misdiagnosed as other diseases in newly diagnosed patients. Although ^18^F-FDG PET/CT showed a higher detection rate (85.0% vs. 50%) for peritoneal metastases than CE-CT, low uptake of ^18^F-FDG (mean SUVmax, 2.7) in these lesions also hinder its capability to clearly depict and accurately assess the severity of the peritoneal lesions. On the contrary, our data demonstrated that ^18^F-FAPI-42 PET/CT had an a much higher uptake of FAP in peritoneal lesions and were more clearly depicted, which thereby contributed to a much higher sensitivity for detection (100%). A similar phenomenon was observed in gastric cancer and colorectal cancer [29, 30]. Due to the higher detection of ^18^F-FAPI-42 PET/CT for peritoneal metastases, the mean PCI score derived from ^18^F-FAPI-42 PET/CT was higher than that derived from ^18^F-FDG PET/ CT (median PCI: 12 vs. 5, *P*<0.001). This suggested that the advantage of ^18^F-FAPI-42 PET/CT for peritoneal metastases could make a positive impact on treatment decision making for appendiceal neoplasms. However, there was still a potential limitation of ^18^F-FAPI-42 PET/CT for the detection of peritoneal metastases in some low aggressive, low-grade appendiceal mucinous neoplasms, which had lower FAPI uptake, similar to the primary tumors.

The results of this study showed that, although ^18^F-FAPI-42 PET/CT had much higher diagnostic accuracy than CE-CT and ^18^F-FDG PET/CT, it did not show particular benefit on treatment management of the appendix in the restaging group. For this group, even if ^18^F-FAPI-42 PET/CT detected more lesions than ^18^F-FDG PET/CT and CE-CT, the stage of the tumor was not changed and the therapy regime remained. Therefore, ^18^F-FAPI-42 PET/CT has less impact on the clinical management of this group of patients than other imaging modalities. However, in patients undergoing initial diagnoses, ^18^F-FAPI-42 PET/CT was much better than CE-CT and ^18^F-FDG PET in detection of the primary tumor. This helped to establish a more accurate diagnosis and resulted in changes to the treatment plan. Therefore, our study highlights that ^18^F-FAPI-42 PET/CT should be recommended for patients with suspected appendiceal neoplasms.

This study has some limitations. First, it was a single-center retrospective study with a small sample size, which was partly caused by the rarity of this tumor. Second, although previous studies have identified FAPI with a high selectivity to FAP positive tumors [35, 36], immunohistochemical staining of FAP must be performed to determine the expression status of FAP in these appendiceal neoplasms. Third, the heterogeneity of PET/CT protocols (e.g. uptake time, dose and use of 2 different PET/CT scanners) in our study may have introduced bias to the SUV measurements.

## Conclusion

The results of this study show that ^18^F-FAPI-42 PET/CT is superior to CE-CT and ^18^F-FDG PET/CT in the visualization and detection of primary and metastatic lesions in patients with appendiceal neoplasms, and plays a potentially important role in the management of disease. Further studies with larger sample sizes are warranted.

## Data Availability

Not applicable.

## Abbreviations

FAPI: Fibroblast-activation protein inhibitor
FDG: Fluorodeoxyglucose
SUVmax: Maximum standardized uptake value
TBR: Target-to-background rate
MTV: Metabolic tumor volume
TLG: Total lesion glycolysis
SRCC: Signet Ring Cell Carcinoma
PCI: Peritoneal cancer index
MIP: Maximal intensity projection
